# Vitamin D and the hepatitis B vaccine response: a prospective cohort study and a randomized, placebo-controlled oral vitamin D_3_ and simulated sunlight supplementation trial in healthy adults

**DOI:** 10.1007/s00394-020-02261-w

**Published:** 2020-05-10

**Authors:** Daniel S. Kashi, Samuel J. Oliver, Laurel M. Wentz, Ross Roberts, Alexander T. Carswell, Jonathan C. Y. Tang, Sarah Jackson, Rachel M. Izard, Donald Allan, Lesley E. Rhodes, William D. Fraser, Julie P. Greeves, Neil P. Walsh

**Affiliations:** 1 College of Human Sciences, Bangor University, Bangor, LL57 2PZ UK; 2 Faculty of Science, Liverpool John Moores University, Liverpool, UK; 3 Beaver College of Health Sciences, Appalachian State University, Boone, USA; 4 Norwich Medical School, University of East Anglia, Norwich, UK; 5 Department of Army Health and Physical Performance Research, Army HQ, Andover, UK; 6 Occupational Medicine, HQ Army Recruiting and Initial Training Command, Upavon, UK; 7 Medical Physics Department, Salford Royal NHS Foundation Trust, and University of Manchester, Manchester Academic Health Science Centre, Manchester, UK; 8 Faculty of Biology, Medicine and Health, University of Manchester, and Dermatology Centre, Salford Royal NHS Foundation Trust, Manchester Academic Health Science Centre, Manchester, UK

**Keywords:** Cholecalciferol, Vitamin D, 25-Hydroxyvitamin D, Hepatitis B, Vaccination, UVB

## Abstract

**Purpose:**

To determine serum 25(OH)D and 1,25(OH)_2_D relationship with hepatitis B vaccination (study 1). Then, to investigate the effects on hepatitis B vaccination of achieving vitamin D sufficiency (serum 25(OH)D ≥ 50 nmol/L) by a unique comparison of simulated sunlight and oral vitamin D_3_ supplementation in wintertime (study 2).

**Methods:**

Study 1 involved 447 adults. In study 2, 3 days after the initial hepatitis B vaccination, 119 men received either placebo, simulated sunlight (1.3 × standard-erythema dose, 3 × /week for 4 weeks and then 1 × /week for 8 weeks) or oral vitamin D_3_ (1000 IU/day for 4 weeks and 400 IU/day for 8 weeks). We measured hepatitis B vaccination efficacy as percentage of responders with anti-hepatitis B surface antigen immunoglobulin G ≥ 10 mIU/mL.

**Results:**

In study 1, vaccine response was poorer in persons with low vitamin D status (25(OH)D ≤ 40 vs 41–71 nmol/L mean difference [95% confidence interval] − 15% [− 26, − 3%]; 1,25(OH)_2_D ≤ 120 vs ≥ 157 pmol/L − 12% [− 24%, − 1%]). Vaccine response was also poorer in winter than summer (− 18% [− 31%, − 3%]), when serum 25(OH)D and 1,25(OH)_2_D were at seasonal nadirs, and 81% of persons had serum 25(OH)D < 50 nmol/L. In study 2, vitamin D supplementation strategies were similarly effective in achieving vitamin D sufficiency from the winter vitamin D nadir in almost all (~ 95%); however, the supplementation beginning 3 days after the initial vaccination did not effect the vaccine response (vitamin D vs placebo 4% [− 21%, 14%]).

**Conclusion:**

Low vitamin D status at initial vaccination was associated with poorer hepatitis B vaccine response (study 1); however, vitamin D supplementation commencing 3 days after vaccination (study 2) did not influence the vaccination response.

**Clinical trial registry number:**

Study 1 NCT02416895; https://clinicaltrials.gov/ct2/show/study/NCT02416895; Study 2 NCT03132103; https://clinicaltrials.gov/ct2/show/NCT03132103.

**Electronic supplementary material:**

The online version of this article (10.1007/s00394-020-02261-w) contains supplementary material, which is available to authorized users.

## Introduction

Discovery of the vitamin D receptor in almost all immune cells, and the many roles vitamin D has in innate and adaptive arms of immunity [1–3], highlight the importance of vitamin D in the regulation of immune responses [4]. As such, avoiding low serum 25-hydroxyvitamin D (25(OH)D) and achieving vitamin D sufficiency (25(OH)D ≥ 50 nmol/L) may be important for the development of vaccine responses and consequently public health [5]. Cell and animal studies indicate that vitamin D may modulate vaccine responses through 1,25-dihydroxyvitamin D (1,25(OH)_2_D) interaction with antigen presentation [6], dendritic cell migration, and the subsequent activation of T and B cell antibody responses [7–9]. Indeed, vitamin D supplementation that corrected wintertime vitamin D status to achieve sufficiency before a tetanus toxoid booster vaccination resulted in higher IgG antibody concentration compared to a placebo [10].

The influence of vitamin D on the development of the hepatitis B vaccination response in humans remains unclear; previous investigations have only studied chronic kidney patients and report conflicting findings [11, 12]. Moreover, the relationship between the biologically active form of vitamin D, 1,25(OH)_2_D, and hepatitis B vaccine is yet to be examined. Hepatitis B vaccination has previously been shown to be influenced by genetics and lifestyle factors [13–15] with 10–15% of adults responding inadequately by producing too few antibodies, as dictated by an anti-hepatitis B surface antigen immunoglobulin G (IgG) concentration of less than 10 mIU/mL [16]. Conversely, those responding to the vaccination with IgG concentration of 10 mIU/mL or more are generally accepted to be protected against infection clinically [16, 17]. Whether vitamin D influences the development of hepatitis B vaccination in healthy adults is unknown, but important to understand given that more than 50% fail to achieve vitamin D sufficiency during winter months [18–20]; and many adults remain unvaccinated because childhood vaccine coverage is ~ 90% or less and routine infant hepatitis B vaccination began only recently in some countries (e.g., UK [21–23]). The hepatitis B vaccination course presents a suitable model to study the influence of vitamin D on the secondary immune response because there is widespread inter-individual variability in the magnitude of the antibody response after the second vaccination, and it is more possible to control prior exposure than with other commonly experienced vaccines (e.g., influenza) [24].

Here, we present results from two studies examining the influence of vitamin D on hepatitis B vaccine response. In these studies, we measured 1,25(OH)_2_D, vitamin D’s biologically active form, and 25(OH)D, which with their respective 4–6-h and 2–3-week half lives can be considered acute and chronic vitamin D status markers, respectively [25]. In study 1, a prospective cohort study of 447 healthy young men and women conducted during all seasons, we examined for the first time serum 1,25(OH)_2_D and 25(OH)D relationship with hepatitis B vaccination in healthy adults. We hypothesized that low serum 1,25(OH)_2_D and 25(OH)D at the time of initial vaccination would be associated with poorer secondary antibody response to hepatitis B vaccination. In study 2, a randomized placebo-controlled trial, we determined the effect of 12-week wintertime vitamin D supplementation on the hepatitis B vaccination response. The supplementation was a unique comparison of simulated sunlight in accordance with recommendations on safe (non-sunburning), low-level sunlight exposure [26], and oral vitamin D_3_ to achieve vitamin D sufficiency (serum 25(OH)D ≥ 50 nmol/L). Vitamin D sufficiency was targeted as maintaining serum 25(OH)D concentration ≥ 50 nmol/L has been recommended for multiple health outcomes [27] by the Institute of Medicine (IOM) and European Food Safety Authority (EFSA) and is achievable using safe doses [19, 20]. The comparison was also made as vitamin D can be obtained from dietary sources but is predominately synthesized by skin exposure to solar ultraviolet (UV) B radiation; UV radiation has a range of vitamin D-dependent and -independent effects on immunity [28, 29]. We hypothesized that vitamin D supplementation that achieves vitamin D sufficiency during winter when vitamin D status is usually low would lead to superior secondary antibody response to hepatitis B vaccination compared to placebo supplementation.

## Methods

The Ministry of Defence (UK) Research Ethics Committee approved these studies, and protocols were conducted in accordance with the Declaration of Helsinki (2013). All participants provided written informed consent.

### Study 1

#### Participant recruitment, inclusion and exclusion criteria

Between June 2014 and November 2015, 1268 men and women who entered the British Army were assessed for eligibility for this prospective cohort study. Eligible participants were ≥ 18 years of age. One thousand one hundred and three recruits volunteered (men from the Infantry Training Centre, Catterick, UK; latitude 54° N, and women from the Army Training Centre, Pirbright, UK; latitude 51° N). Participants were excluded from the final analysis if they failed the initial medical assessment, followed an atypical hepatitis B vaccination schedule (the first two vaccine doses were not administered within 4 weeks of each other), or did not provide a blood sample to assess the secondary hepatitis B vaccine response. Participants were also excluded from statistical analysis if their medical records documented previous exposure to hepatitis B vaccination; or, if this was later confirmed by measurable antibody titers against hepatitis B surface antigen detected in baseline samples (anti-HBs titers > 0 mIU/mL). The baseline demographics, anthropometrics, and lifestyle behaviors for the 447 participants included in the final analysis are summarized in Table 1 (Supplemental Table 1 includes details of the larger recruited sample).

**Table 1:** Study 1 baseline participant demographics, anthropometrics, lifestyle behaviors, sleep, mood and all-cause illness in cohorts recruited across seasons

	All *n* = 447	Winter	Spring	Summer	Autumn
		*n* = 88	*n* = 63	*n* = 115	*n* = 181
Demographics					
Age (years)	21.7 ± 3.0	21.5 ± 3.0	22.1 ± 3.2	21.9 ± 3.0	21.5 ± 3.1
Ethnicity, Caucasian [*n* (%)]	434 (97)	85 (97)	62 (98)	109 (96)	178 (98)
Anthropometrics					
Height (m)	1.73 ± 0.08	1.73 ± 0.09	1.71 ± 0.09	1.75 ± 0.08	1.71 ± 0.08
Body mass (kg)	71.8 ± 10.8	72.1 ± 11.3	70.8 ± 10.8	74.0 ± 10.7	70.5 ± 10.5
BMI (kg/m^2^)	24.0 ± 2.7	23.9 ± 2.8	24.2 ± 2.7	24.1 ± 2.6	23.9 ± 2.7
Lifestyle behaviors					
Alcohol user, [*n* (%)]	376 (88)	76 (93)	50 (82)	99 (87)	151 (88)
Smoker, [*n* (%)]	259 (58)	53 (61)	38 (60)	71 (62)	97 (54)
Sleep night before initial vaccination					
Duration (h)	6.4 ± 0.8	6.3 ± 0.7	6.4 ± 0.5	6.3 ± 0.9	6.6 ± 0.9
Quality (very poor = 1 to very good = 4)	1.7 ± 0.8	1.7 ± 0.8	1.6 ± 0.7	1.8 ± 0.8	1.6 ± 0.8
Contraception (*n* = 138)^a^					
None, [*n* (%)]	36 (26)	7 (19)	4 (11)	5 (14)	20 (56)
COCP, [*n* (%)]	50 (36)	9 (18)	15 (30)	5 (10)	21 (42)
POP, [*n* (%)]	9 (7)	2 (22)	2 (22)	1 (11)	4 (45)
Injection, [*n* (%)]	8 (6)	2 (25)	0 (0)	1 (12)	5 (63)
Implant, [*n* (%)]	35 (25)	9 (26)	6 (17)	5 (14)	15 (43)
Mood before initial vaccination^b^					
Vigor	8.4 ± 3.0	8.5 ± 3.0	7.3 ± 3.1	8.6 ± 2.8	8.7 ± 3.1
Anger	0.9 ± 1.6	0.6 ± 1.1	0.7 ± 1.5	1.0 ± 1.6	0.9 ± 1.7
Tension	4.8 ± 3.4	4.1 ± 3.1	4.7 ± 3.7	4.3 ± 3.1	5.3 ± 3.5
Confusion	2.3 ± 2.4	2.4 ± 2.8	1.8 ± 1.9	2.5 ± 2.4	2.3 ± 2.5
Depression	0.7 ± 1.6	0.6 ± 1.1	0.6 ± 2.0	0.7 ± 1.3	0.8 ± 1.7
Fatigue	4.2 ± 3.0	3.6 ± 2.9	4.3 ± 3.3	4.2 ± 2.8	4.4 ± 3.0
All-cause illness [*n* (%)]^c^	71 (16)	8 (9)*	10 (16)	10 (9)*	43 (24)

Values presented as mean ± SD, unless otherwise stated

*COCP* combined oral contraceptive pill, *POP* progesterone-only pill

*P < 0.05 lower than autumn

^a^Female contraception data collected from a female specific questionnaire (*n* = 37 excluded from final data analysis)

^b^Greater scores indicate a greater feeling of the mood (maximum per mood = 20)

^c^Physician diagnosed cases of respiratory and gastrointestinal tract infection

#### Procedures

**Fig. 1:**
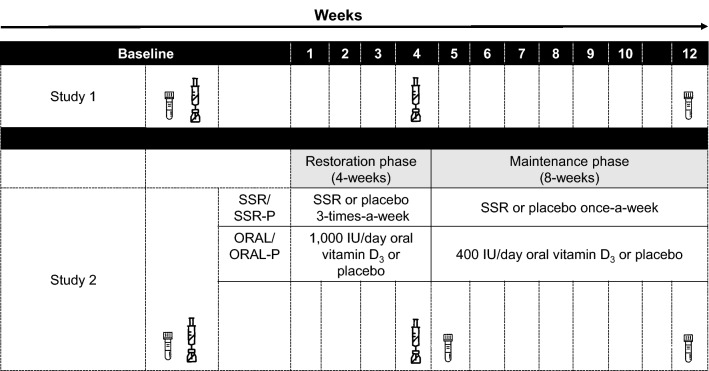
Schematic of study 1 and 2 procedures. Study 1 investigated the influence of vitamin D status at the time of the initial hepatitis B vaccination on the secondary antibody response to hepatitis B vaccination. Study 2 investigated the effect of vitamin D supplementation by solar-simulated radiation (SSR), oral vitamin D_3_ (ORAL), or placebo (SSR-P or ORAL-P) after the initial hepatitis B vaccination on secondary hepatitis B vaccine response. Needle and bottle icon represents hepatitis B vaccination doses. Blood tube icon represents when blood samples were obtained for serum 25(OH)D, 1,25(OH)_2_D and hepatitis B antibody titer measurements

Before participants commenced Basic Military training, they completed an initial medical assessment. During the initial medical assessment, participants received their first 20-μg dose of recombinant hepatitis B vaccine into the deltoid muscle (Engerix-B, Smithkline Beecham Pharmaceuticals, Uxbridge, UK) and a venous blood sample was collected for the determination of hepatitis B antibody titer, serum 25(OH)D and 1,25(OH)_2_D concentrations (Fig. 1). At the initial medical assessment, we also collected baseline measures of participant demographics (e.g., ethnicity) and anthropometrics; height and body mass were assessed in light clothing with shoes removed by stadiometer and digital platform scale, respectively (SECA 703, Birmingham, UK). Lifestyle factors previously shown to influence the vaccination response were also assessed by questionnaire; including alcohol and smoking use, sleep and mood [13–15]. To assess sleep duration and quality the night before vaccination participants completed a questionnaire based on the procedures of Prather et al. [15]. Sleep duration was calculated as the number of hours and minutes elapsed between the time they reported going to sleep and the time they reported waking. Sleep quality was reported on a scale from 1 = very poor to 4 = very good. Before receiving their initial hepatitis B vaccination, participants also completed a Brunel mood scale (BRUMS) [30], which measures 6 moods (vigor, anger, tension, confusion, depression, fatigue). Each mood is assessed by 4 items scored from 0 = not at all to 4 = extremely and, therefore, the maximum score per mood is 20, with greater scores indicating a greater feeling of the mood. In line with the typical hepatitis B vaccination schedule, participants received a second 20-μg hepatitis B vaccine dose 1 month after the first. A second venous blood sample was collected 8 weeks after the second hepatitis B vaccine dose (3 months after the first hepatitis B vaccine dose) to determine secondary serum hepatitis B antibody titers, the primary outcome measure. The serum hepatitis B antibody titer (anti-HBs) was assessed as this is the routine serological test to determine if a person has been successfully vaccinated against hepatitis B [16]. We focused on the antibody response to the second vaccination because there is widespread inter-individual variability in the magnitude of antibody response following the second vaccination of the typical three-dose series [24]. This variability is in distinct contrast with the antibody response to the first vaccination, when < 10% of individuals have detectable levels of antibody, or the third, when the majority of individuals have mounted maximal antibody responses, respectively [15]. ‘All-cause illness’ consisting of physician diagnosed cases of upper and lower respiratory tract infection and gastrointestinal infection were also retrieved from medical records for the period of basic training.

### Study 2

#### Participant recruitment and exclusion criteria

Healthy men were recruited in a double-blind randomized, placebo-controlled trial upon entering the British Army Combat Infantryman’s Course, Catterick, UK during January and February of 2016 and 2017, when ambient UVB is negligible at UK latitudes (50–60° N), and serum 25(OH)D is at a seasonal low. Eligible participants were ≥ 17 years of age and had passed the initial medical assessment; had no history of skin cancer, photosensitivity, or lupus erythematosus; and had sun-reactive skin types I–IV [31]. Participants were excluded for the same reasons as in study 1, plus current consumption of vitamin D in dietary supplements; use of a sunbed or travel to a sunny climate 3 months before the study.

#### Experimental procedures

Participants had the same baseline assessments and hepatitis B vaccination schedule as study 1 (Fig. 1). Following this, we block randomized participants within their platoons to one of the four intervention groups: (1) solar-simulated radiation (SSR); (2) solar-simulated radiation placebo (SSR-P); (3) oral vitamin D_3_ (ORAL); or (4) oral placebo (ORAL-P). Block randomization by randomizer.org resulted in an equal distribution of intervention groups within each platoon, and ensured any differences in training conditions between platoons did not influence the study outcomes. An independent researcher completed the randomization and investigators were blind to the randomization until statistical analyses were completed. The interventions began 3 days after the initial hepatitis B vaccine dose. The intervention strategy for the SSR and ORAL groups was to restore and then maintain vitamin D sufficiency (serum 25(OH)D ≥ 50 nmol/L) as recommended by Institute of Medicine (IOM) and the European Food Safety Authority (EFSA) [19, 20]. Participants completed a 4-week restoration phase, necessary because serum 25(OH)D was at its winter nadir, followed by an 8-week maintenance phase (Fig. 1). Blood samples were obtained at baseline, and after 5 and 12 weeks for the determination of serum 25(OH)D and 1,25(OH)_2_D (Fig. 1). Vitamin D from solar UV radiation exposure was estimated in weeks 4 and 11 using polysulphone badges and from the diet in week 12 using a food frequency questionnaire [32]. On completion of the study, participants completed an ‘exit survey’, which required them to guess the intervention they thought they had been receiving.

### Simulated sunlight intervention

In accordance with guidelines on safe, low-level sunlight exposure for vitamin D synthesis [26], and as described previously to achieve vitamin D sufficiency (serum 25(OH)D ≥ 50 nmol/L) in the majority of white-skinned persons [33], those assigned to the SSR intervention were exposed three-times-a-week, during the restoration phase to an investigator-controlled constant UV radiation dose using a whole-body irradiation cabinet (Hapro Jade, Kapelle, The Netherlands) fitted with Arimed B fluorescent tubes (Cosmedico, Stuttgart, Germany). The fluorescent tubes emitted a UV radiation spectrum similar to sunlight (*λ* 290–400 nm; 95% UVA 320–400 nm, 5% UVB 290–320 nm) that was characterized by a spectroradiometer (USB2000 +, Ocean Optics BV, Duiven, The Netherlands) radiometrically calibrated with traceability to UK national standards. During each exposure, participants received a 1.3 standard erythemal dose (SED), and wore shorts and a T-shirt to expose ~ 40% of skin surface area. This dose is equivalent to ~ 15-min midday summer sun exposure in northern England (latitude 53.5° N) [33] and taking account of pre-vitamin D irradiance at different latitudes, can be related to exposure times at other world locations [34]. For example, the equivalent exposure time in Philadelphia, Pennsylvania, USA (40° N) would be ~ 12 min; and that for Oslo, Norway (60° N) would be ~ 18 min. During the maintenance phase, we exposed SSR participants to the same 1.3 × SED dose only once-a-week: pilot investigations confirmed the required dose to maintain sufficiency (serum 25(OH)D ≥ 50 nmol/L). A constant SSR dose was maintained during the study by monitoring irradiance using a spectroradiometer (USB2000 +, Ocean Optics BV) and adjusting for any decrease in measured irradiance emitted by increasing exposure time (mean duration of SSR exposures was 229 ± 17 s). We controlled the exposure time using an electronic timer. Participants undergoing SSR-P treatment received the same number of intervention exposures each week and the exposure duration as SSR except the irradiation cabinet fluorescent tubes were covered with transparent UV radiation blocking film (DermaGard UV film, SunGard, Woburn, Massachusetts, USA) [35] in a manner invisible to the participants and experimenters. Spectroradiometry confirmed that the UV radiation blocking film was effective at preventing transmission of 99.9% of UV radiation.

### Oral vitamin D_3_ intervention

Participants receiving the ORAL intervention consumed a daily vitamin D_3_ supplement containing 1000 IU and 400 IU vitamin D_3_ during the restoration phase and maintenance phase, respectively (Pure Encapsulations, Sudbury, Massachusetts, USA) [35]. The restoration dose (1000 IU/day) was based on previous predictive modeling to achieve serum 25(OH)D ≥ 50 nmol/L [36], and pilot investigations that showed it achieved similar serum 25(OH)D concentrations to SSR; and was less than the tolerable upper intake recommended by IOM and EFSA [19, 20]. The ORAL maintenance dose (400 IU/day) was in accordance with recommendations [19]. For 12 weeks, ORAL-P participants consumed a daily oral cellulose placebo capsule, identical in size, shape and color to the vitamin D_3_ capsules (Almac Group, County Armagh, UK). Independent analysis found the vitamin D_3_ content of the 1000 and 400 IU capsules to be 1090 and 460 IU, respectively and confirmed that the placebo did not contain vitamin D (NSF International Laboratories, Ann Arbor, Michigan, USA).

### Biochemical analyses (study 1 and 2)

Whole blood samples were collected by venepuncture from an antecubital vein into plain vacutainer tubes (Becton–Dickinson, Oxford, UK) and left to clot for 1 h. Subsequently, samples were centrifuged at 1500*g* for 10 min at 4 °C and the serum aliquoted into Eppendorf tubes before being immediately frozen at − 80 °C for later analysis. Baseline and secondary serum antibody titers were determined using a hepatitis B antibody enzyme-linked immunoassay kit (DiaSorin, Saluggia, Italy). The intra-assay coefficient of variation was 4.9% (study 1) and 5.9% (study 2). Total serum 25(OH)D was measured with high-pressure liquid chromatography–tandem mass spectrometry [37]; and serum 1,25(OH)_2_D using the DiaSorin LIAISON XL 1,25(OH)_2_D chemiluminescent immunoassay (Stillwater, Minnesota, USA) method. Analyses were performed in a Vitamin D External Quality Assurance Scheme certified laboratory (Bioanalytical Facility, University of East Anglia, Norwich, UK).

### Statistical analysis

Secondary antibody titers have a non-normal distribution and, therefore, in line with the previous research [17], we categorized the development of secondary antibody response to the hepatitis B vaccine as the percentage of participants with serum antibody titer response to hepatitis B ≥ 10 mIU/mL. Those participants with anti-HBs titers ≥ 10 mIU/mL were categorized as vaccine ‘responders’; whilst, those with antibody titers < 10 mIU/mL were categorized as vaccine ‘non-responders’ [17]. Further, those responding to the vaccination with anti-HBs titers of 10 mIU/mL or more are generally accepted to be protected against infection clinically [16, 17]. The sample size estimation for study 1 and 2 was calculated as a minimum of 152, using the anticipated difference in hepatitis B vaccine responder rate of 20% (Cohen’s *h* = 0.4; small–medium effect size) between individuals displaying low and high vitamin D status [11], with a type 1 error (one tailed) of 5%, and a power of 80%. For study 1, we used Chi square analysis to compare the percentage of hepatitis B vaccine responders in those with IOM-defined vitamin D sufficient status (serum 25(OH)D ≥ 50 nmol/L) compared to those with serum < 50 nmol/L. However, as there is no consensus to the optimal vitamin D threshold for immune function [18, 38], we conducted Kruskal–Wallis tests to compare the percentage of hepatitis B vaccine responders across 25(OH)D, 1,25(OH)_2_D and 24,25(OH)_2_D terciles. One-way ANOVA and Kruskal–Wallis tests were used, where appropriate, to compare serum vitamin D (25(OH)D and 1,25(OH)_2_D), percentage of participants displaying serum 25(OH)D ≥ 50 nmol/L and the percentage of hepatitis B vaccine responders across seasons. Independent *t* test, Chi square, one-way ANOVA and Kruskal–Wallis tests were used, where appropriate, to compare demographic, anthropometric, alcohol and smoking use, sleep, mood, contraception use in women, ‘all-cause illnes’ data across seasons and between participants with serum 25(OH)D ≥ 50 nmol/L and < 50 nmol/L. For study 2, Kruskal–Wallis was used to compare the percentage of secondary hepatitis B vaccine responders after SSR, ORAL, SSR-P and ORAL-P. In addition, the percentage of secondary hepatitis B vaccine responders was compared between vitamin D supplementation (SSR and ORAL combined) and placebo groups (SSR-P and ORAL-P combined) using Chi square analysis. Serum 25(OH)D and 1,25(OH)_2_D were compared between vitamin D and placebo groups using mixed-model ANOVA (4 group (SSR, ORAL, SSR-P and ORAL-P) × 3 time points (baseline, week 5 and 12) and 2 group (SSR and ORAL combined, SSR-P and ORAL-P) × 3 time points. Post hoc comparisons were conducted using Bonferroni corrected *t* tests. Chi square tests were conducted to compare the percentage of participants displaying total serum 25(OH)D ≥ 50 nmol/L at baseline, week 5 and week 12 between vitamin D and placebo groups. Independent samples *t* test, Mann–Whitney *U* and Chi square tests were used to compare demographic, anthropometric, alcohol and smoking use, sleep, and mood data between vitamin D and placebo supplement groups. All statistical analyses were completed using SPSS Statistics 22 (IBM, Armonk, New York, USA).

## Results

### Study 1

#### Participant flow

**Fig. 2:**
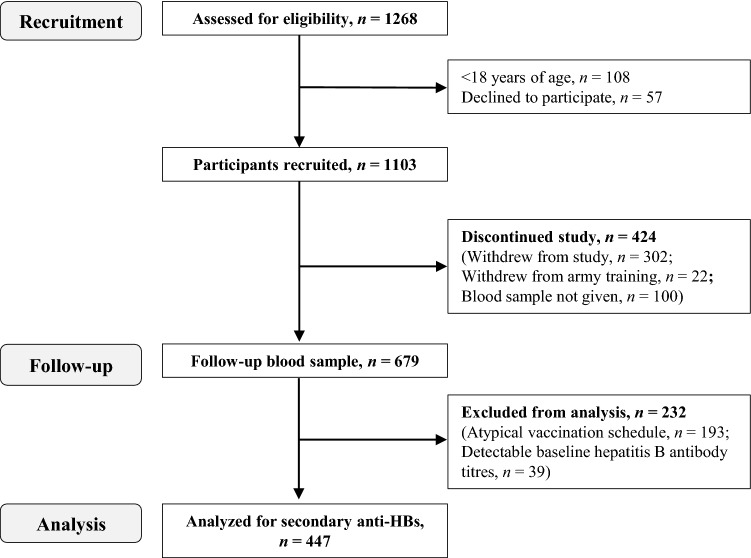
Flow diagram indicating the numbers of participants assessed for eligibility, recruited, available at follow-up, and analyzed as part of Study 1. *Anti-HBs* antibodies against hepatitis B antigen

A total of 1103 men and women were recruited from June 2014 to November 2015. Participants began the study throughout the year: 20% in winter (December–February), 14% in spring (March–May), 26% in summer (June–August), and 40% in autumn (September–November). Participant flow, drop-out and exclusion before biochemical and statistical analysis are summarized in Fig. 2. There was no significant difference in demographics, anthropometrics, lifestyle behaviors, sleep, mood, contraception use, or all-cause illness between participants included and excluded in the final analysis (Supplemental Table 2).

#### Vitamin D and secondary hepatitis B vaccine response

**Fig. 3:**
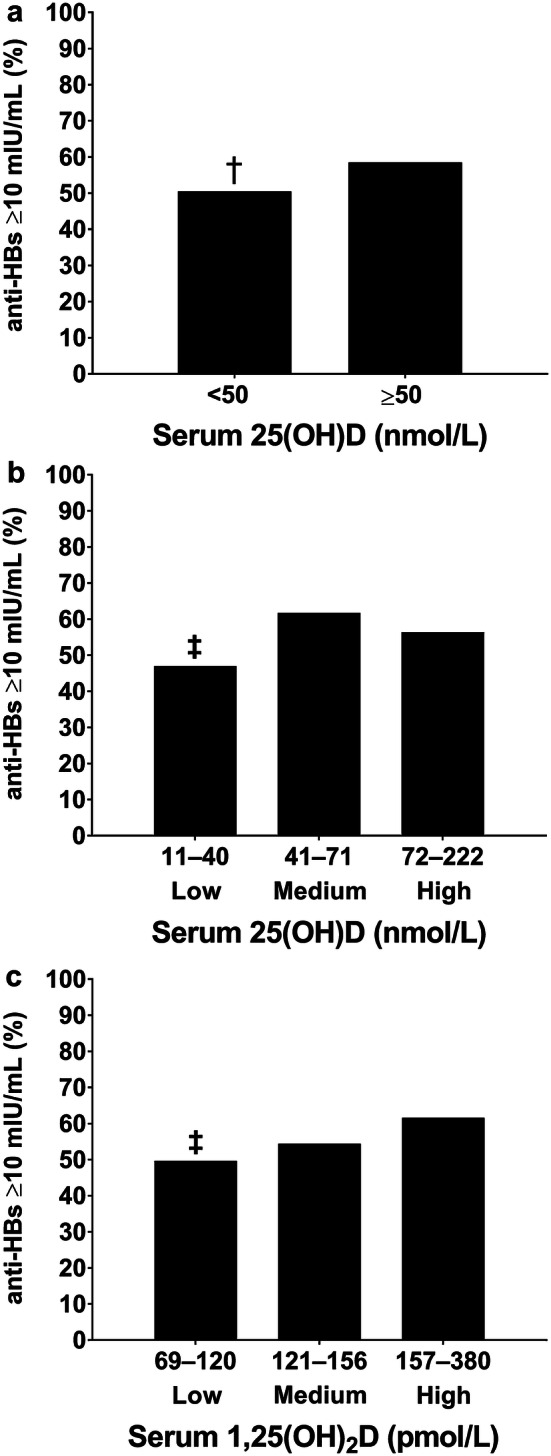
Secondary hepatitis B vaccine response in those with serum 25(OH)D < 50 nmol/L (*n* = 194) and serum 25(OH)D ≥ 50 nmol/L (*n* = 253 adults, (**a**), and low, medium and high serum 25(OH)D (**b**), *n* = 447) and low, medium and high 1,25(OH)_2_D terciles (**c**), *n* = 444). ^†^*P *< 0.1, lower percentage of secondary hepatitis B vaccination responders (anti-HBs ≥ 10 mIU/mL) in participants with 25(OH)D < 50 nmol/L than vitamin D-sufficient participants. ^‡^*P* < 0.05, lower percentage of secondary hepatitis B vaccination responders (anti-HBs ≥ 10 mIU/mL) in low 25(OH)D and 1,25(OH)_2_D terciles compared to medium 25(OH)D and high serum 1,25(OH)_2_D terciles

At the time of the initial vaccination, 43% of participants had serum 25(OH)D < 50 nmol/L, 26% were vitamin D insufficient (serum 25(OH)D 30–50 nmol/L), and 17% were vitamin D deficient (serum 25(OH)D < 30 nmol/L). Only 1 participant presented with severe vitamin D deficiency (serum 25(OH)D < 12.5 nmol/L). Fewer participants tended to respond to the hepatitis B vaccination who had 25(OH)D < 50 nmol/L than those who were vitamin D sufficient at the time of initial vaccination (50% vs 58%, mean difference [95% confidence interval], − 8% [− 17%, 1%], *P *= 0.09, *h* = 0.16, Fig. 3a). Moreover, hepatitis B vaccine response was poorer in those with serum 25(OH)D ≤ 40 nmol/L (mean 30 ± 7 nmol/L) compared to participants with 25(OH)D between 41 and 71 nmol/L (mean 56 ± 9 nmol/L) at the time of initial vaccination (mean difference [95% confidence interval], − 15% [− 26%, − 3%], *P *= 0.01, Fig. 3b). Fewer participants were also hepatitis B vaccine responders when they presented with low serum 1,25(OH)_2_D compared to participants who presented with high serum 1,25(OH)_2_D at the time of initial vaccination (50% vs 62%, mean difference [95% confidence interval], − 12% [− 24%, − 1%,], *P *< 0.05, *h* = 0.24, Fig. 3c). Furthermore, fewer participants were hepatitis B vaccine responders when they presented with combined low 1,25(OH)_2_D and 25(OH)D compared to combined medium–high 25(OH)D and 1,25(OH)_2_D (43% vs 65%, mean difference [95% confidence interval], − 22% [− 39%, − 5%], *P *= 0.01). No differences were observed between those who presented with low serum 24,25(OH)D compared to participants who presented with high serum 24,25(OH)D at the time of initial vaccination (52% vs 60%, mean difference [95% confidence interval], − 8% [20%, 3%], *P *= 0.14).

There were no differences between participants with 25(OH)D ≥ 50 nmol/L and < 50 nmol/L in demographics, anthropometrics, lifestyle behaviors, sleep, mood, contraception use, or all-cause illness before the initial hepatitis B vaccination (Table 2). Anthropometrics, lifestyle behaviors, sleep, mood and all-cause illness also did not predict vaccine response (*P *> 0.05). Additionally, contraception use did not influence the vaccine response (*P *> 0.05, e.g., none vs oral contraception, 68% vs 62% mean difference [95% confidence interval], 6% [− 9, 21%]). Further regression analysis controlling for BMI, smoking, alcohol, sleep and mood indicated that vitamin D-sufficient men, but not women, were 1.8 times more likely to be vaccine responders than those with serum 25(OH)D < 50 nmol/L (OR [95% confidence interval], men 1.8 [1.0, 3.2] and women 0.8 [0.4, 1.7]). Serum 25(OH)D, 1,25(OH)_2_D, 24,25(OH)_2_D, vitamin D sufficiency and hepatitis B response were lower in men than women (*P *< 0.05, men vs women: 25(OH)D, 56 ± 30 vs 69 ± 32 nmol/L; 1,25(OH)_2_D, 126 ± 32 vs 165 ± 43 pmol/L; 24,25(OH)_2_D, 4.4 ± 2.8 vs 6.5 ± 3.7 nmol/L; vitamin D sufficiency, 49% vs 69%; hepatitis B response, 49% vs 65%).

**Table 2:** Study 1 baseline participant demographics, anthropometrics, lifestyle behaviors, sleep, mood and all-cause illness in those with serum 25(OH)D < 50 nmol/L and $\ge$ 50 nmol/L

	Serum 25(OH)D	
	< 50 nmol/L *n* = 194	≥ 50 nmol/L *n* = 253
Demographics		
Men [*n* (%)]	139 (72)	133 (53)
Women [*n* (%)]	55 (28)	120 (47)
Age (years)	21.3 ± 2.9	22.0 ± 3.2
Ethnicity, Caucasian [*n* (%)]	186 (96)	248 (98)
Anthropometrics		
Height (m)	1.74 ± 0.08	1.71 ± 0.09
Body mass (kg)	73.4 ± 10.8	70.1 ± 10.7
BMI (kg/m^2^)	24.2 ± 2.8	23.9 ± 2.6
Lifestyle behaviors		
Alcohol user, [*n* (%)]	167 (86)	209 (83)
Smoker, [*n* (%)]	122 (63)	137 (54)
Sleep night before initial vaccination		
Duration (h)	6.6 ± 0.7	6.3 ± 0.9
Quality (very poor = 1 to very good = 4)	1.7 ± 0.7	1.7 ± 0.8
Contraception (*n* = 138)^a^		
None, [*n* (%)]	14 (33)	22 (23)
COCP, [*n* (%)]	10 (23)	40 (43)
POP, [*n* (%)]	2 (5)	7 (7)
Injection, [*n* (%)]	4 (9)	4 (4)
Implant, [*n* (%)]	13 (30)	22 (23)
Mood before initial vaccination^b^		
Vigor	8.4 ± 3.1	8.4 ± 3.0
Anger	0.8 ± 1.4	0.9 ± 1.6
Tension	4.7 ± 3.5	4.8 ± 3.3
Confusion	2.5 ± 2.6	2.2 ± 2.3
Depression	0.8 ± 1.8	0.7 ± 1.4
Fatigue	4.2 ± 3.0	4.3 ± 3.0
All-cause illness [*n* (%)]^c^	29 (15)	42 (17)

Values presented as mean ± SD unless otherwise stated. There were no significant differences between vitamin D sufficient (25(OH)D ≥ 50 nmol/L) and those participants with 25(OH)D < 50 nmol/L in demographic, anthropometrics, lifestyle behaviors, sleep, mood or all-cause illness before the initial hepatitis B vaccination at baseline

*COCP* combined oral contraceptive pill, *POP* progesterone-only pill

^a^Female contraception data collected from a female specific questionnaire (*n* = 37 excluded from final data analysis)

^b^Greater scores indicate a greater feeling of the mood (maximum per mood = 20)

^c^Physician diagnosed cases of respiratory and gastrointestinal tract infection

#### Seasonal variation in vitamin D and hepatitis B vaccine response

**Fig. 4:**
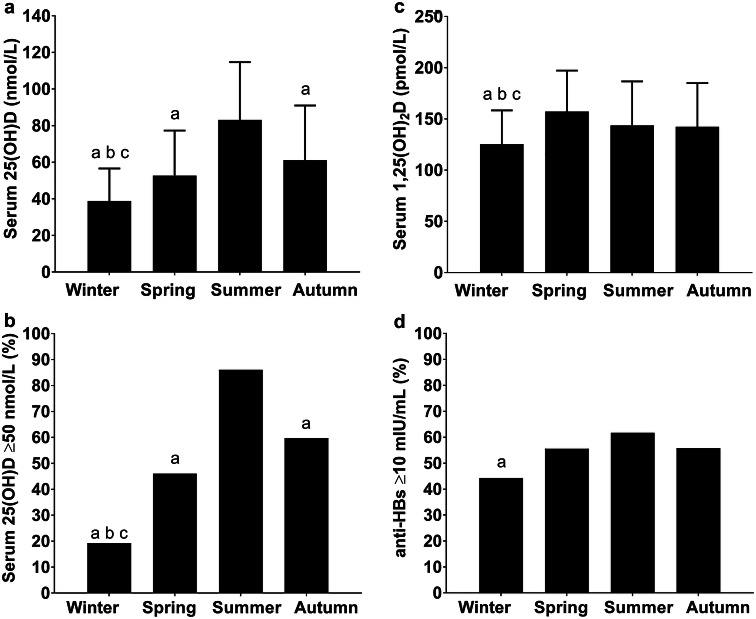
Seasonal variation in serum 25(OH)D (**a**), percentage of participants categorized as vitamin D sufficient (25(OH)D ≥ 50 nmol/L; (**b**), serum 1, 25(OH)_2_D (**c**), and percentage of secondary hepatitis B vaccination responders (anti-HBs ≥ 10 mIU/mL; (**d**) in 447 healthy, young men (*n *= 272) and women (*n *= 175) residing in the UK. **a**, **c** Data are mean ± SD. **b**, **d** Are percentages represented by vertical bars. **a** Lower than summer (*P *< 0.05). **b** Lower than autumn (*P *< 0.05). **c** Lower than spring (*P *< 0.05)

Serum 25(OH)D, 1,25(OH)_2_D and vitamin D sufficiency (25(OH)D ≥ 50 nmol/L) were lower in winter than spring, summer and autumn (*P *< 0.05, Fig. 4a–c). In winter, 81% participants had 25(OH)D < 50 nmol/L (Fig. 4b) with 32% of participants vitamin D deficient (serum 25(OH)D < 30 nmol/L). The percentage of hepatitis B vaccine responders was also lower in winter than summer (44% vs 62%, mean difference [95% confidence interval], − 18% [− 31%, − 3%], *P *< 0.05, *h* = 0.36, Fig. 4d). With the exception of all-cause illness, participants recruited in the different seasons were similar as indicated by no differences in demographic, anthropometrics, lifestyle behaviors, sleep, mood or use of contraception in women before the initial hepatitis B vaccination (Table 1). Similar seasonal variations in serum 24,25(OH)_2_D were also observed with winter serum 24,25(OH)_2_D contractions lower than summer and autumn (*P *< 0.05, winter 2.9 ± 2.2 nmol/L, spring 4.2 ± 2.8 nmol/L, summer 6.5 ± 3.2 nmol/L, autumn 5.9 ± 3.4 nmol/L).

### Study 2

#### Participant flow and blinding

**Fig. 5:**
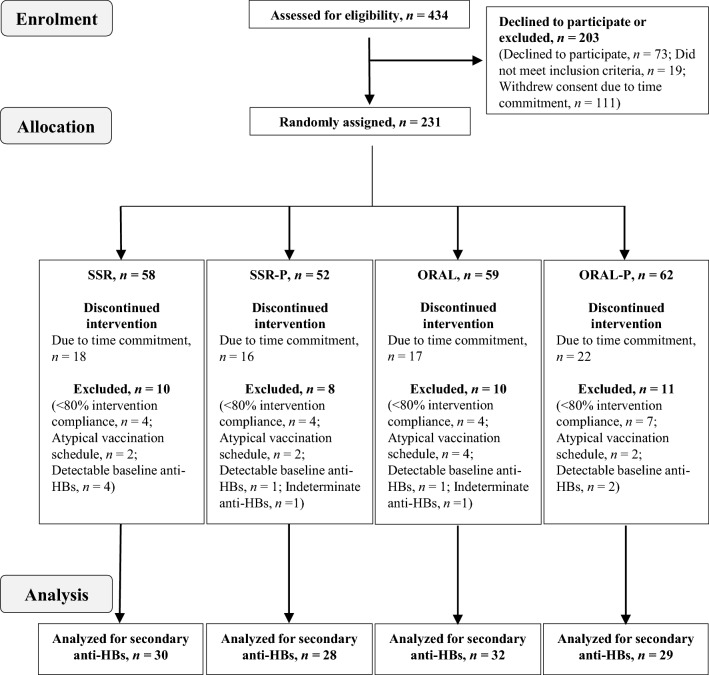
CONSORT flow diagram indicating the numbers of participants assessed, recruited, randomly assigned, and analyzed as part of study 2. *Anti-HBs* antibodies against hepatitis B antigen, *vitamin D = SSR* solar-simulated radiation, *ORAL* oral vitamin D_3_, *Placebo = SSR-P* solar-simulated radiation placebo, *ORAL-P* oral placebo

Two hundred and thirty-one men were assigned to the interventions in January and February of 2016 and 2017. The study ended after reaching its scheduled date of closure. Participant flow, drop-out and exclusion before biochemical and statistical analysis are summarized in Fig. 5. There was no significant difference in demographics, anthropometrics, lifestyle behaviors, sleep or mood between participants included and excluded in the final analysis (Supplemental Table 3). There were no adverse events reported relating to vitamin D or placebo supplementation. Participants were sufficiently blinded to the intervention since only 35% correctly guessed their allocated group, 30% were incorrect, and 35% said they did not know whether they had received an active (SSR and ORAL) or placebo (SSR-P and ORAL-P) intervention.

#### The influence of low-level simulated sunlight and oral vitamin D_3_ on vitamin D status

**Fig. 6:**
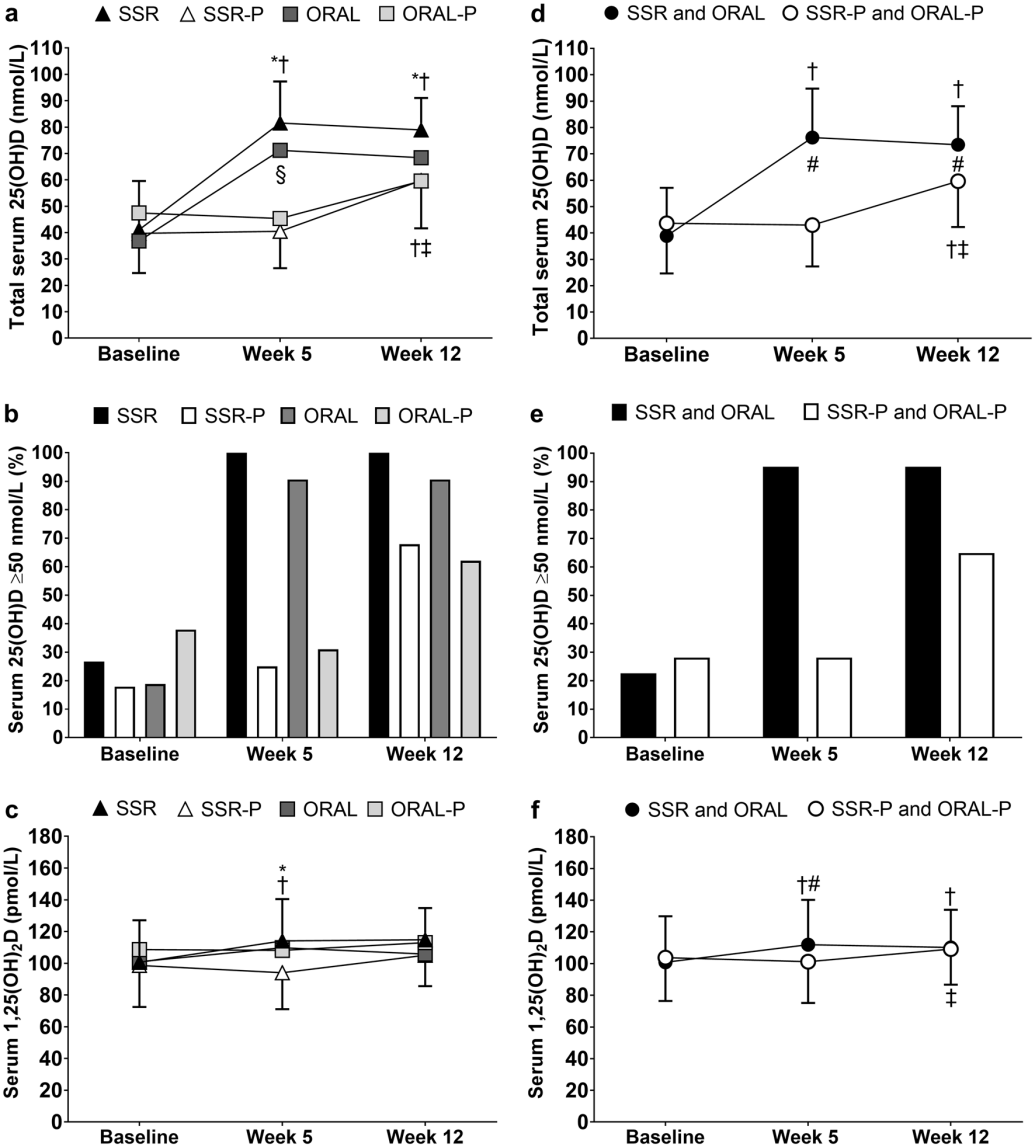
Serum 25(OH)D (**a**, **d**), percentage of participants categorized as vitamin D sufficient (serum 25(OH)D ≥ 50 nmol/L, (**b**, **e**), serum 1,25(OH)_2_D **c**, **f** in response to 12 weeks of vitamin D supplementation by solar-simulated radiation (SSR) and oral vitamin D_3_ (ORAL). **a**–**c** Show comparisons of individual vitamin D and placebo supplementation groups (SSR, SSR-P, ORAL and ORAL-P). **d**–**f** Show combined vitamin D supplementation (SSR and ORAL) vs combined placebo (SSR-P and ORAL-P) groups. ^†^*P* < 0.05, greater than baseline. ^‡^*P* < 0.05, greater than week 5. *P < 0.05, greater than SSR-P. ^§^*P* < 0.05, greater than ORAL-P. ^#^*P* < 0.05, greater than combined SSR-P and ORAL-P. Data are mean ± SD (**a**, **c**, **d f**) and vertical bars represent percentages (**b**, **e**)

At baseline, 75% of the volunteers had 25(OH)D < 50 nmol/L, 45% were vitamin D insufficient (serum 25(OH)D 30–50 nmol/L), and 30% were vitamin D deficient (serum 25(OH)D < 30 nmol/L). Only 1 participant presented with severe vitamin D deficiency (serum 25(OH)D < 12.5 nmol/L). There was no difference between vitamin D and placebo supplementation groups’ demographics, anthropometrics, lifestyle behaviors, sleep, mood (Table 3), or vitamin D status (Fig. 6, *P* > 0.05). There were also no differences in these variables between combined vitamin D and placebo supplemented groups (Supplemental Table 4 and Fig. 6). During the 12-week intervention, daily sunlight exposure was low, as expected considering the latitude and time of year [39], with similar sunlight exposure (0.22 ± 0.33 SED/day; *P* > 0.05) and dietary vitamin D intake (112 ± 84 IU/day, *P* > 0.05) in vitamin D and placebo supplement groups.

**Table 3:** Study 2 baseline participant demographics, anthropometrics, lifestyle behaviors, sleep and mood in solar-simulated radiation (SSR), SSR placebo (SSR-P) oral vitamin D_3_ (ORAL) and oral placebo (ORAL-P) supplemented groups

	SSR *n* = 30	SSR-P *n* = 28	ORAL *n* = 32	ORAL-P *n* = 29
Demographics				
Age (years)	21.5 ± 3.1	21.7 ± 3.4	20.9 ± 2.7	21.4 ± 3.0
Ethnicity (Caucasian) [*n* (%)]	29 (97)	28 (100)	32 (100)	29 (100)
Skin type (I, II, III, IV) [*n* (%)]^a^	3 (10), 8 (27), 14 (46), 5 (17)	2 (7), 10 (36), 13 (46), 3 (11)	3 (9), 11 (34), 13 (41), 5 (16)	2 (7), 9 (31), 15 (52), 3 (10)
Anthropometrics				
Height (m)	1.78 ± 0.05	1.77 ± 0.05	1.78 ± 0.07	1.78 ± 0.06
Body mass (kg)	76.7 ± 11.6	76.8 ± 9.7	75.7 ± 12.3	77.5 ± 10.8
BMI (kg/m^2^)	24.3 ± 3.3	24.4 ± 2.8	24.9 ± 2.8	24.9 ± 2.8
Lifestyle behaviors				
Alcohol user [*n* (%)]	23 (77)	22 (79)	26 (81)	23 (77)
Smoker [*n* (%)]	17 (57)	16 (57)	17 (53)	11 (38)
Sleep night before initial vaccination				
Duration (h)	6.2 ± 0.8	5.9 ± 1.4	5.8 ± 1.5	5.8 ± 1.8
Quality (very poor = 1 to very good = 4)	2.9 ± 0.7	2.8 ± 0.7	2.8 ± 0.7	2.8 ± 0.7
Mood before initial vaccination^b^				
Vigor	8.0 ± 3.4	9.0 ± 2.9	7.1 ± 2.9	8.2 ± 3.2
Anger	1.0 ± 1.8	1.5 ± 2.5	1.2 ± 2.0	0.7 ± 1.6
Tension	3.0 ± 2.2	3.6 ± 3.4	3.2 ± 3.3	2.6 ± 2.1
Confusion	2.6 ± 3.2	2.5 ± 2.9	1.7 ± 2.1	1.5 ± 1.9
Depression	0.7 ± 1.8	1.4 ± 2.7	0.6 ± 1.6	0.3 ± 0.6
Fatigue	3.6 ± 2.7	4.9 ± 3.2	4.1 ± 3.5	4.1 ± 3.1

Values presented as mean ± SD unless otherwise stated. There were no significant differences between supplemented groups in demographics, anthropometrics, lifestyle behaviors, sleep or mood before the initial hepatitis B vaccination at baseline (*P* > 0.05)

^a^Skin types are based on Fitzpatrick scale [31]

^b^Greater scores indicate a greater feeling of the mood (maximum per mood = 20)

The vitamin D supplementation was successful in achieving vitamin D sufficiency and maintaining serum 25(OH)D concentrations, so that at weeks, 5 and 12 serum 25(OH)D concentrations in the vitamin D supplementation groups were higher than the placebo groups (*P* < 0.05, Fig. 6d). By week 5, 95% of participants in the vitamin D supplementation groups were vitamin D sufficient (25(OH)D ≥ 50 nmol/L, Fig. 6e). There was no difference in serum 25(OH)D or percentage of participants achieving vitamin D sufficiency between vitamin D supplementation groups (*P* > 0.05). Serum 1,25(OH)_2_D was similar in all groups at baseline (*P *> 0.05) and increased with supplementation (*P *< 0.05, Fig. 6f), with greater responses in the vitamin D supplementation groups compared to the placebo groups at week 5 (*P *< 0.05). There was no difference between groups at week 12 (*P *> 0.05) because 1,25(OH)_2_D increased from weeks 5 to 12 in placebo groups (*P *< 0.05). Serum 24,25(OH)_2_D responded similarly to supplementation as serum 25(OH)D, so that at weeks 5 and 12, serum 24,25(OH)_2_D concentrations in the vitamin D supplementation groups were higher than the placebo groups (*P* < 0.05, Supplemental Table 5).

#### The influence of simulated sunlight and oral vitamin D_3_ on secondary hepatitis B vaccine response

**Fig. 7:**
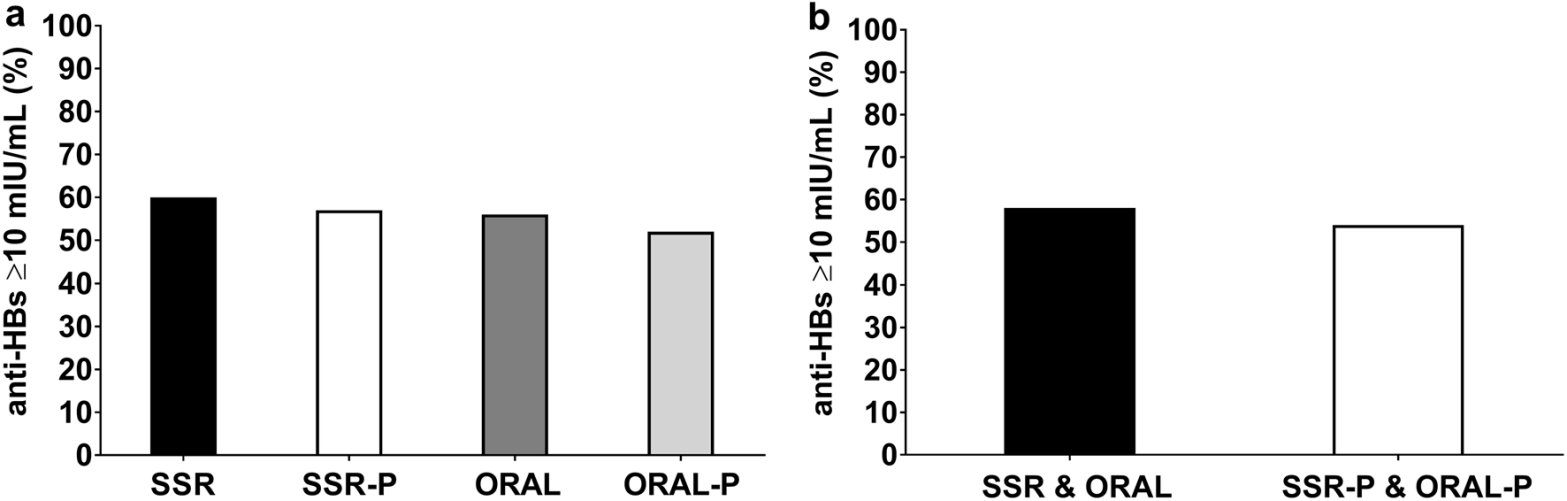
Percentage of participants categorized as secondary hepatitis B vaccine responders (anti-HBs ≥ 10 mIU/mL, (**a**, **b**) after 12 weeks of vitamin D supplementation by solar-simulated radiation (SSR) and oral vitamin D_3_ (ORAL). **a** Compares individual vitamin D and placebo supplementation groups (SSR, SSR-P, ORAL and ORAL-P). **b** Shows combined vitamin D supplementation (SSR and ORAL) vs combined placebo (SSR-P and ORAL-P) groups. There was no difference in vaccine response between individual vitamin D and placebo supplementation groups (**a** SSR 60%, SSR-P 57%, ORAL 56%, ORAL-P 52%, *P *> 0.05) or between combined vitamin D and placebo groups (**b** SSR and ORAL 58% vs SSR-P and ORAL-P 54%, *P *> 0.05)

Vitamin D supplementation beginning 3 days after the initial vaccination did not influence the secondary antibody response as the percentage of secondary hepatitis B vaccine responders was similar among the vitamin D and placebo groups (SSR 60%, SSR-P 57%, ORAL 56%, ORAL-P 52%, *P *> 0.05, Fig. 7a). Analyses comparing combined vitamin D to placebo also revealed no effect of vitamin D supplementation on secondary hepatitis B vaccine response (SSR and ORAL vs SSR-P and ORAL-P, 58% vs 54%, mean difference [95% confidence interval], 4% [− 21%, 14%], *P *> 0.05, *h* = 0.08, Fig. 7b). Furthermore, a secondary analysis including only men who had 25(OH)D < 50 nmol/L at baseline also revealed no effect of vitamin D supplementation on secondary hepatitis B vaccine response (*P *> 0.05).

## Discussion

We determined the influence of vitamin D on the development of the hepatitis B vaccination in healthy adults. In study 1, vitamin D status (25(OH)D and 1,25(OH)_2_D) at the time of initial vaccination influenced the subsequent secondary hepatitis B vaccine response: low vitamin D status was associated with poorer hepatitis B vaccine response (Fig. 3). Analysis controlling for demographic, anthropometric, and lifestyle factors revealed that vitamin D sufficient men, but not women, were nearly 2 times more likely to be responders to the hepatitis B vaccine than those with serum 25(OH)D of < 50 nmol/L. These differences may be explained by lower serum 25(OH)D and 1,25(OH)_2_D in men and a lower proportion of men achieving vitamin D sufficiency compared to women. Indeed, the hepatitis B vaccine response was poorer in men than women. Furthermore, hepatitis B vaccine response was associated with seasonal alterations in serum 25(OH)D and 1,25(OH)_2_D, with poorer hepatitis B vaccine responses in winter than summer (Fig. 4d). The findings of study 1 indicated a possible immunomodulatory role of vitamin D in the development of hepatitis B vaccine response. Given these findings, and the high prevalence of serum 25(OH)D < 50 nmol/L during winter (81% of persons had serum 25(OH)D < 50 nmol/L in study 1), in study 2 we examined the effect of winter vitamin D supplementation on hepatitis B vaccine response. Study 2, a randomized, placebo-controlled trial, involved a unique comparison of safe, simulated, casual skin sunlight exposure and oral vitamin D_3_ supplementation specifically designed to achieve vitamin D sufficiency. Contrary to our hypothesis, and despite achieving and maintaining IOM and EFSA defined vitamin D sufficiency in 95% of participants (Fig. 6), vitamin D supplementation beginning 3 days after the initial hepatitis B vaccination did not influence the hepatitis B vaccine response (Fig. 7).

The divergent findings of studies 1 and 2 are contrary to our hypothesis; however, they are consistent with animal and human studies that have identified it is the early (within 24 h), rather than later, stages of orchestrating the development of immunity that are most sensitive to intervention [40, 41]. Indeed, vitamin D, and specifically 1,25(OH)_2_D, may influence the hepatitis B vaccine response by stimulating antigen presenting cells, which are pivotal for the initial capturing, processing and presenting of the antigen at the site of vaccination [42, 43]. In animal models, it has been observed that locally produced 1,25(OH)_2_D induced migration of dendritic cells from the site of vaccination to non-draining lymphoid organs, where they can stimulate antigen-specific T and B cells to mount a strong and persistent antibody response to diphtheria vaccination [7, 8]. Co-administration of 1,25(OH)_2_D with trivalent influenza vaccine in mice was shown to enhance both mucosal and systemic specific antibody response [44, 45], and highlights vitamin D as a potential vaccine adjuvant. In addition, previous research in humans has shown higher IgG antibodies in response to tetanus toxoid vaccination after 9 weeks of vitamin D supplementation compared to a placebo group [10], which lends further support to the notion of vitamin D as a potential adjuvant for vaccines more generally.

In both studies, we were unable to collect an additional blood sample after the third, and final, hepatitis B vaccine dose; therefore, it remains to be determined whether vitamin D influences the final development of the hepatitis B vaccine response. As non-responders to initial vaccine dose tend to be poorer responders to subsequent doses [15], it is reasonable to hypothesize that persons low in vitamin D at the initial hepatitis B vaccination are more likely to be vaccine non-responders after the full hepatitis B vaccine course (Fig. 3). Future studies should, however, confirm the influence of vitamin D status at the time of initial vaccination on final antibody status after the full hepatitis B vaccine course. Study 1 was a prospective cohort study, and it is, therefore, possible that factors other than vitamin D may explain the associations observed between vitamin D, season and the hepatitis B vaccine response. Previously, body mass index, mood, sleep and lifestyle (alcohol and smoking use) have been shown to influence the hepatitis B vaccination response [13–15]. Further, seasonal alterations in infectious disease and compromised host immunity might influence seasonal alterations in hepatitis B vaccination independent of vitamin D status [46]. A strength of our studies is that we took account of these factors and showed they were similar across the seasons (Table 1), and between persons who were vitamin D sufficient and not (Table 2), and supplementation groups (Table 3). Furthermore, all-cause illness, a marker of host immunity (Tables 1, 2), and living conditions were also similar. These similarities strengthen the argument that vitamin D, rather than another factor, is responsible for observed association with hepatitis B vaccination in study 1. Nonetheless, future randomized control studies using similar supplementation methods as study 2 that improve vitamin D status before the initial vaccination would verify whether vitamin D status at the time of initial vaccination is important in the development of the hepatitis B response.

The objective of these original studies was to explore the influence of vitamin D status on the hepatitis B vaccination response, with the interventions designed to achieve vitamin D sufficiency including a 4-week period of low-level SSR (12 exposures) followed by 8 weeks of maintenance SSR (8 exposures). While vitamin D synthesis is the major established health benefit of UVR, the latter has immunomodulatory (both suppressive and augmenting) effects, which may be mediated through vitamin D-dependent and -independent pathways [28, 29]. Thus, a previous human study of contrasting design examined for a possible effect of prior acute higher-level UVR exposure (UVB therapy lamps; daily exposures given at the individual’s sunburn threshold for 5 days) on the first hepatitis B vaccination response [47]. The investigators did not relate their findings to vitamin D status. They found no overall impact of UVR on cellular (lymphocyte stimulation test) or humoral (antibody titre) response to hepatitis B surface antigen, despite the UVR regime being adequate to reduce other immune responses, i.e., contact hypersensitivity and natural killer cell activity. Further analysis found individual difference in susceptibility, with a reduced vaccination response observed in those individuals with a minor variant of IL-1beta polymorphism; prevalence of the variant is low and further studies are suggested [48].

In combination with findings in elderly chronic kidney disease patients [11], our findings in healthy adults highlight the potential importance of preventing low vitamin D status at the time of the initial vaccination for the adequate development of the hepatitis B vaccination. Future research is merited to confirm the influence of vitamin D on the hepatitis B vaccination response in infants and the elderly, who are at greater risk of poor vitamin D status than healthy young adults [49], and because the hepatitis B vaccination is mandatory during infancy in several countries [21, 22]. This does not reduce the impact of the current studies findings as many adults remain unvaccinated because childhood vaccine coverage is ~ 90% or less and routine infant hepatitis B vaccination began only recently in some countries (e.g., UK [21–23]). Adult vaccination is recommended for persons at increased risk of exposure to bodily fluids such as health care professionals, patients, and those traveling to areas of the world where hepatitis B is widespread, e.g., sub-Saharan Africa, east and southeast Asia and the Pacific Islands [16]. The 1,25(OH)_2_D findings from study 1 also provide a mechanism by which maintaining vitamin D sufficiency and high 1,25(OH)_2_D may be important for vaccine immunogenicity beyond hepatitis B. As more than 50% fail to achieve vitamin D sufficiency during winter months [24–26], future research to further understand the role of vitamin D on vaccination more broadly is warranted. The 8% difference in hepatitis B vaccination response between people who were vitamin D sufficient and 25(OH)D < 50 nmol/L, and the 18% difference between winter and summer (Figs. 3a, 4d) are comparable with the effects on the hepatitis B vaccine response shown for other lifestyle factors, e.g., smoking, obesity and poor sleep [13, 15]. Of particular clinical interest, the winter vaccine response (44% anti-HBs titers ≥ 10 mIU/mL) was poorer than typically expected after two hepatitis B vaccine doses (50–90%: Fig. 4) [50]. Therefore, rather than restoring vitamin D sufficiency from its winter nadir, as in study 2, we suggest maintaining year-round vitamin D sufficiency, and where necessary preventing the decline in the end of summer serum 25(OH)D by commencing vitamin D supplementation in late summer or early autumn and continuing until spring (~ 6 months). To maintain the end of summer serum 25(OH)D, individuals should aim to achieve current IOM and EFSA vitamin D dietary intake recommendations [19, 20]. We achieved this in study 2 with a daily 400 IU oral vitamin D_3_ dose (Fig. 6). Oral vitamin D_3_ supplementation is recommended in the autumn and winter because unlike simulated sunlight, there is no time burden for an individual; no requirement for bulky irradiation cabinets; and oral vitamin D_3_ supplementation is effective regardless of sun reactive skin type [51]. Further, even very low sub-sunburn UVR doses were recently shown to cause skin cell DNA damage in easy-burning skin types [52]. Low-level sunlight exposure, as used in study 2, may, however, provide benefits to human health in addition to vitamin D synthesis, and this is an active area of research [29].

## Conclusions

In a prospective cohort study of 447 healthy adults (study 1), vitamin D sufficiency was rare during the UK winter, and fewer people responded to the hepatitis B vaccination than during the summer. In study 1, poorer vitamin D status (serum 1,25(OH)_2_D ≤ 120 pmol/L and 25(OH)D ≤ 40 nmol/L) at the time of initial vaccination was associated with fewer healthy adults responding to the hepatitis B vaccine. In a subsequent randomized control trial (study 2), vitamin D supplementation (oral or via simulated sunlight exposure) that began 3 days after the initial vaccination, and achieved vitamin D sufficiency within 5 weeks, did not influence the hepatitis B vaccination response. Randomized control trials that manipulate vitamin D status before the initial vaccination are warranted to confirm the influence of vitamin D status at the time of initial vaccination on the hepatitis B vaccine response. In accordance with the findings of the prospective cohort study (study 1), avoiding low vitamin D status at the time of the initial hepatitis B vaccination, by maintaining year-round vitamin D sufficiency, might be recommended to optimize the response to hepatitis B vaccination. This is particularly important for persons that rely on effective vaccination prophylaxis such as health care professionals and patients regularly exposed to bodily fluids.

## Electronic supplementary material

Below is the link to the electronic supplementary material.

Supplementary material 1 (DOCX 44 kb)
