# Supplementary material for: Vitamin D and the hepatitis B vaccine response: a prospective cohort study and a randomized, placebo-controlled oral vitamin D3 and simulated sunlight supplementation trial in healthy adults
Source: Eur J Nutr. 2020 May 10;60(1):475–91. doi: 10.1007/s00394-020-02261-w (PMC7867563; doi:10.1007/s00394-020-02261-w)
Supplement: Supplementary file 1 — Supplementary material 1 (DOCX 44 kb) [file 394_2020_2261_MOESM1_ESM.docx]

**Supplemental Table 1**. Study 1 baseline participant demographics, anthropometrics, lifestyle behaviors, sleep, mood and all cause illness in cohorts recruited across the seasons.

|  | All | *Winter* | *Spring* | *Summer* | *Autumn* |
| --- | --- | --- | --- | --- | --- |
|  | *n* = 1003 | *n* = 182 | *n* = 183 | *n* = 296 | *n* = 342 |
| *Demographics* |  |  |  |  |  |
| Age (years) | 21.8 ± 5.7 | 21.4 ± 3.0 | 21.6 ± 2.9 | 21.8 ± 6.0 | 22.1 ± 7.5 |
| Ethnicity, Caucasian [*n* (%)] | 952 (95) | 175 (96) | 174 (95) | 276 (93) | 327 (96) |
|  |  |  |  |  |  |
| *Anthropometrics* |  |  |  |  |  |
| Height (m) | 1.73 ± 0.08 | 1.74 ± 0.08 | 1.73 ± 0.08 | 1.75 ± 0.08 | 1.72 ± 0.09 |
| Body mass (kg) | 71.8 ± 10.5 | 71.2 ± 10.4 | 72.0 ± 10.9 | 73.7 ± 10.0 | 70.4 ± 10.6 |
| BMI (kg/m^2^) | 23.8 ± 3.1 | 23.6 ± 2.8 | 24.1 ± 2.7 | 24.0 ± 2.5 | 23.5 ± 3.7 |
|  |  |  |  |  |  |
| *Lifestyle behaviors* |  |  |  |  |  |
| Alcohol user, [*n* (%)] | 832 (83) | 155 (85) | 142 (78) | 254 (86) | 281 (82) |
| Smoker, [*n* (%)] | 579 (58) | 112 (62) | 98 (54) | 185 (62) | 184 (54) |
|  |  |  |  |  |  |
| *Sleep night before initial vaccination* |  |  |  |  |  |
| Duration (h) | 6.4 ± 0.8 | 6.2 ± 0.6 | 6.4 ± 0.5 | 6.2 ± 0.9 | 6.6 ± 0.8 |
| Quality (very poor =1 to very good =4) | 1.7 ± 0.8 | 1.7 ± 0.8 | 1.6 ± 0.8 | 1.8 ± 0.8 | 1.7 ± 0.8 |
|  |  |  |  |  |  |
| *Contraception (n =259)^1^* |  |  |  |  |  |
| None, [*n* (%)] | 68 (26) | 12 (18) | 12 (18) | 11 (16) | 33 (48) |
| COCP, [*n* (%)] | 82 (32) | 12 (15) | 21 (26) | 12 (15) | 37 (45) |
| POP, [*n* (%)] | 23 (9) | 7 (30) | 5 (22) | 1 (4) | 10 (43) |
| Injection, [*n* (%)] | 21 (8) | 3 (14) | 3 (14) | 3 (14) | 12 (57) |
| Implant, [*n* (%)] | 65 (25) | 16 (25) | 13 (20) | 6 (9) | 30 (46) |
|  |  |  |  |  |  |
|  |  |  |  |  |  |
| *Mood before initial vaccination* |  |  |  |  |  |
| Vigor | 8.4 ± 3.1 | 8.3 ± 3.0 | 8.3 ± 3.3 | 8.6 ± 3.0 | 8.3 ± 3.0 |
| Anger | 1.0 ± 1.8 | 0.8 ± 1.4 | 0.9 ± 1.9 | 1.1 ± 1.8 | 1.1 ± 1.9 |
| Tension | 4.7 ± 3.4 | 4.4 ± 3.2 | 4.3 ± 3.5 | 4.3 ± 3.2 | 5.3 ± 3.5 |
| Confusion | 2.4 ± 2.6 | 2.3 ± 2.6 | 2.0 ± 2.4 | 2.5 ± 2.5 | 2.5 ± 2.6 |
| Depression | 0.9 ± 1.9 | 0.8 ± 1.6 | 0.9 ± 2.1 | 0.9 ± 1.9 | 1.0 ± 1.9 |
| Fatigue | 4.7 ± 3.4 | 4.2 ± 3.2 | 4.3 ± 3.2 | 4.6 ± 3.1 | 4.4 ± 3.1 |
| *All cause illness* [*n* (%)] | 173 (17) | 23 (13)^*^ | 37 (20) | 38 (13)^*^ | 75 (22) |

Values presented as mean ± SD, unless otherwise stated. ^*^ *P* < 0.05 lower than autumn. COCP, combined oral contraceptive pill, POP, progesterone-only pill. All-cause illness data consisted of physician diagnosed cases of respiratory and gastrointestinal tract infection. The 1,003 participants are those recruited minus 100 participants that did not give an initial blood sample, and subsequently did not have the characteristics listed in this table assessed. ^1^Female contraception data collected from a female specific questionnaire (n = 56 excluded from final data analysis).

**Supplemental Table 2**. A comparison of baseline participant demographics, anthropometrics, lifestyle behaviors, sleep, mood and all cause illness between participants included and excluded from the final Study 1 analysis

|  | Included  *n* = 447 | Excluded  *n* = 556 |
| --- | --- | --- |
| *Demographics* |  |  |
| Men [*n* (%)] | 272 (61) | 400 (72) |
| Women [*n* (%)] | 175 (39) | 156 (28) |
| Age (years) | 21.7 ± 3.0 | 21.9 ± 7.2 |
| Ethnicity, Caucasian [*n* (%)] | 433 (97) | 519 (93) |
|  |  |  |
| *Anthropometrics* |  |  |
| Height (m) | 1.73 ± 0.08 | 1.74 ± 0.08 |
| Body mass (kg) | 71.8 ± 10.8 | 71.9 ± 10.2 |
| BMI (kg/m^2^) | 24.0 ± 2.7 | 23.7 ± 3.0 |
|  |  |  |
| *Lifestyle behaviors* |  |  |
| Alcohol user, [*n* (%)] | 376 (84) | 457 (82) |
| Smoker, [*n* (%)] | 259 (58) | 320 (57) |
|  |  |  |
| *Sleep night before initial vaccination* |  |  |
| Duration (h) | 6.4 ± 0.8 | 6.4 ± 0.7 |
| Quality (very poor =1 to very good =4) | 1.7 ± 0.8 | 1.7 ± 0.8 |
|  |  |  |
| *Contraception (n = 259)^1^* |  |  |
| None, [*n* (%)] | 36 (26) | 32 (26) |
| COCP, [*n* (%)] | 50 (36) | 32 (26) |
| POP, [*n* (%)] | 9 (6) | 14 (12) |
| Injection, [*n* (%)] | 8 (6) | 13 (10) |
| Implant, [*n* (%)] | 35 (25) | 30 (25) |
| *Mood before initial vaccination* |  |  |
| Vigor | 8.4 ± 3.0 | 8.4 ± 3.1 |
| Anger | 0.9 ± 1.6 | 1.2 ± 1.9 |
| Tension | 4.8 ± 3.4 | 4.6 ± 3.5 |
| Confusion | 2.3 ± 2.4 | 2.4 ± 2.6 |
| Depression | 0.7 ± 1.6 | 1.0 ± 2.1 |
| Fatigue | 4.2 ± 3.0 | 4.6 ± 3.2 |
| *All cause illness* [*n* (%)] | 75 (17) | 98 (18) |
| Values presented as mean ± SD unless otherwise stated. COCP, combined oral contraceptive pill, POP, progesterone-only pill. ^*^ *P* < 0.05 lower than participants included in final analysis. Excluded contains participants that withdrew from the study, withdrew from army training, had atypical vaccination schedules or detectable anti-HBs at baseline, minus 100 participants that did not give an initial blood sample, and subsequently did not have the characteristics listed in this table assessed. ^1^Female contraception data collected from a female specific questionnaire (n = 56 excluded from final data analysis). | | |

**Supplemental Table 3**. A comparison of baseline participant demographics, anthropometrics, lifestyle behaviors, sleep, and mood between participants included and excluded from the final Study 2 analysis

|  | Included  *n* = 119 | Excluded  *n* = 112 |
| --- | --- | --- |
| *Demographics* |  |  |
| Age (years) | 21.3 ± 3.0 | 22.4 ± 9.5 |
| Ethnicity, Caucasian [*n* (%)] | 118 (99) | 109 (97) |
| Skin type (I, II, III, IV) [*n* (%)] | 10 (8), 38 (32),  55 (47), 16 (13) | 6 (5), 31 (28),  54 (48), 21 (19) |
| *Anthropometrics* |  |  |
| Height (m) | 1.77 ± 0.06 | 1.77 ± 0.07 |
| Body mass (kg) | 76.7 ± 11.2 | 76.4 ± 10.6 |
| BMI (kg/m^2^) | 24.4 ± 3.0 | 24.2 ± 2.9 |
| *Lifestyle behaviors* |  |  |
| Alcohol user, [*n* (%)] | 94 (79) | 96 (86) |
| Smoker, [*n* (%)] | 61 (51) | 71 (63) |
|  |  |  |
| *Sleep night before initial vaccination* |  |  |
| Duration (h) | 6.1 ± 0.9 | 6.0 ± 1.2 |
| Quality (very poor =1 to very good =4) | 2.8 ± 0.7 | 2.7 ± 0.8 |
|  |  |  |
| *Mood before initial vaccination* |  |  |
| Vigor | 8.1 ± 3.1 | 7.2 ± 3.3 |
| Anger | 1.1 ± 2.0 | 1.0 ± 1.9 |
| Tension | 3.1 ± 2.8 | 2.7 ± 2.7 |
| Confusion | 2.1 ± 2.6 | 1.5 ± 1.9 |
| Depression | 0.8 ± 1.9 | 0.6 ± 1.8 |
| Fatigue | 4.1 ± 3.1 | 3.6 ± 2.7 |
| Values presented as mean ± SD unless otherwise stated. There was no significant difference in demographics, anthropometrics, lifestyle behaviors, sleep or mood between participants included and excluded in the final analysis. | | |

**Supplemental Table 4**. Study 2 baseline participant demographics, anthropometrics, lifestyle behaviors, sleep and mood in combined vitamin D and placebo supplemented groups

|  |  | | Vitamin D  (SSR and ORAL)  *n* = 62 | | Placebo  (SSR-P and ORAL-P)  *n* = 57 |
| --- | --- | --- | --- | --- | --- |
| *Demographics* | |  | |  |  |
| Age (years) | |  | | 21.2 ± 2.9 | 21.5 ± 3.2 |
| Ethnicity (Caucasian) [*n* (%)] | |  | | 61 (98) | 57 (100) |
| Skin type (I, II, III, IV) [*n* (%)] | |  | | 6 (10), 19 (31),  27 (43), 10 (16) | 4 (7), 19 (33),  28 (49), 6 (11) |
| *Anthropometrics* | |  | |  |  |
| Height (m) | |  | | 1.77 ± 0.06 | 1.77 ± 0.06 |
| Body mass (kg) | |  | | 76.2 ± 11.9 | 77.3 ± 10.4 |
| BMI (kg/m^2^) | |  | | 24.2 ± 3.2 | 24.6 ± 2.7 |
|  | |  | |  |  |
| *Lifestyle behaviors* | |  | |  |  |
| Alcohol user [*n* (%)] | |  | | 49 (79) | 45 (79) |
| Smoker [*n* (%)] | |  | | 34 (55) | 27 (47) |
| *Sleep night before initial vaccination* | |  | |  |  |
| Duration (h) | |  | | 6.0 ± 1.3 | 5.9 ± 1.6 |
| Quality (very poor = 1 to very good = 4) | |  | | 2.8 ± 0.7 | 2.7 ± 0.7 |
| *Mood before initial vaccination* | |  | |  |  |
| Vigor | |  | | 7.6 ± 3.3 | 8.6 ± 3.1 |
| Anger | |  | | 1.1 ± 1.9 | 1.1 ± 2.1 |
| Tension | |  | | 3.1 ± 2.8 | 3.1 ± 2.8 |
| Confusion | |  | | 1.0 ± 1.1 | 1.3 ± 2.3 |
| Depression | |  | | 0.7 ± 1.6 | 0.8 ± 1.9 |
| Fatigue | |  | | 3.8 ± 3.1 | 4.5 ± 3.2 |

Values presented as mean ± SD unless otherwise stated. There were no significant differences between vitamin D (SSR and ORAL) and placebo (SSR-P & ORAL-P) supplemented groups in demographics, anthropometrics, lifestyle behaviors, sleep or mood before the initial hepatitis B vaccination at baseline (*P* > 0.05)

**Supplemental table 5**. Serum 24,25(OH)_2_D response to 12-weeks of vitamin D supplementation by solar simulated radiation (SSR), oral vitamin D_3_ (ORAL) and placebo groups (SSR-P, ORAL-P).

|  | SSR | SSR-P | ORAL | ORAL-P | Vitamin D  (SSR and ORAL) | Placebo  (SSR-P and ORAL-P) |
| --- | --- | --- | --- | --- | --- | --- |
| *Week* |  |  |  |  |  |  |
| Baseline | 2.5 ± 1.8 | 2.3 ± 1.5 | 2.3 ± 1.5 | 3.2 ± 1.8^‡^ | 2.4 ± 1.7 | 2.8 ± 1.7 |
| 5 | 5.3 ± 1.6^†*^ | 2.2 ± 1.3 | 4.7 ± 1.6^†§^ | 2.7 ± 1.5 | 5.0 ± 1.6^†#^ | 2.5 ± 1.4 |
| 12 | 5.7 ± 1.5^†*^ | 3.4 ± 1.5^†,‡^ | 4.8 ± 1.2^†§^ | 3.5 ± 1.3^‡^ | 5.2 ± 1.4^†#^ | 3.5 ± 1.4^†‡^ |
| Values are presented as mean ± SD. SSR-P, SSR placebo group, ORAL-P, ORAL placebo group. † *P <* 0.05 greater than baseline, ‡ *P <* 0.05 greater than week 5. * *P* < 0.05, greater than SSR-P. § *P* < 0.05, greater than ORAL-P. # *P <* 0.05 greater than combined SSR-P and ORAL-P. | | | | | | |
